# EEG as a Functional Marker of Nicotine Activity: Evidence From a Pilot Study of Adults With Late-Life Depression

**DOI:** 10.3389/fpsyt.2021.721874

**Published:** 2021-12-23

**Authors:** Alexander C. Conley, Alexandra P. Key, Warren D. Taylor, Kimberly M. Albert, Brian D. Boyd, Jennifer N. Vega, Paul A. Newhouse

**Affiliations:** ^1^Department of Psychiatry, Center for Cognitive Medicine, Vanderbilt University Medical Center, Nashville, TN, United States; ^2^Vanderbilt Department of Hearing and Speech Sciences, Vanderbilt University Medical Center, Nashville, TN, United States; ^3^Department of Veterans Affairs Medical Center, Geriatric Research, Education and Clinical Center, Tennessee Valley Healthcare System, Nashville, TN, United States

**Keywords:** nicotine, EEG, late-life depression, beta power, event-related oscillations

## Abstract

Late-life depression (LLD) is a debilitating condition that is associated with poor response to antidepressant medications and deficits in cognitive performance. Nicotinic cholinergic stimulation has emerged as a potentially effective candidate to improve cognitive performance in patients with cognitive impairment. Previous studies of nicotinic stimulation in animal models and human populations with cognitive impairment led to examining potential cognitive and mood effects of nicotinic stimulation in older adults with LLD. We report results from a pilot study of transdermal nicotine in LLD testing whether nicotine treatment would enhance cognitive performance and mood. The study used electroencephalography (EEG) recordings as a tool to test for potential mechanisms underlying the effect of nicotine. Eight non-smoking participants with LLD completed EEG recordings at baseline and after 12 weeks of transdermal nicotine treatment (NCT02816138). Nicotine augmentation treatment was associated with improved performance on an auditory oddball task. Analysis of event-related oscillations showed that nicotine treatment was associated with reduced beta desynchronization at week 12 for both standard and target trials. The change in beta power on standard trials was also correlated with improvement in mood symptoms. This pilot study provides preliminary evidence for the impact of nicotine in modulating cortical activity and improving mood in depressed older adults and shows the utility of using EEG as a marker of functional engagement in nicotinic interventions in clinical geriatric patients.

## Introduction

Late Life Depression (LLD) or geriatric depression, is the occurrence of major depressive disorder in adults over 60 years of age (1). LLD is associated with impaired cognitive performance (2). A major issue for the treatment of adults with LLD is that patients have a poor response rate to current antidepressants (3). A recent meta-analysis of studies assessing antidepressant treatment response in the elderly demonstrated a 50.7% response rate of patients with LLD (4). Poor treatment response has been associated with executive dysfunction (5, 6), as well as other cognitive domains including processing speed, language and episodic memory (7). As a response to these difficulties in ensuring treatment response in older adults, new strategies are being developed to address this problem. It is also important to understand mechanistically how such treatments may contribute to the clinical response. One of the more promising techniques for use in rapidly evaluating the cortical effects of potential treatments in LLD is electroencephalography (EEG), which has the advantages of being both cost-effective and with high temporal resolution, which allows for the examination of direct markers of the cortical activity response to treatment.

EEG has been utilized in adults with LLD to identify the differences in cortical activity compared to healthy older adults. Previous research found that patients with LLD display slower information processing with longer P300 latencies to auditory stimuli, as well as diminished inhibitory processing compared to healthy controls (8, 9). LLD patients have also been observed to exhibit greater slow wave power at rest compared to age-matched controls (8, 10). As shown in younger adults, EEG may serve as a predictor of response to antidepressant treatment (11, 12). A six-week study of paroxetine in men with major depressive disorder (MDD) showed that in addition to improved mood symptoms, chronic treatment led to a reduction in alpha power and an increase in delta, theta and beta power, particularly at bilateral frontal areas (13). Baseline EEG markers, including N1 amplitudes to oddball trials and resting frontal theta power have also been associated with greater subsequent reductions of depressive symptoms over 8 weeks of treatment (14).

A novel treatment with preliminary evidence for benefit to both mood and cognitive performance in LLD is nicotine (15). Both preclinical and clinical trials of nicotine have observed improvements in mood following nicotinic stimulation (16–19). Beyond mood, nicotine appears to improve the cognitive performance of healthy adults, specifically on tasks assessing attention (20, 21). These results have also been seen in older cognitively impaired patients, with a 6-month trial of transdermal nicotine in patients with Mild Cognitive Impairment (MCI) demonstrating improved sustained attention performance compared to placebo (22).

EEG may serve as a useful measure for the effects of nicotine on brain function. Nicotine in healthy adults has been shown to reduce slow wave power and increase power in both alpha and beta bands (23–26). Nicotine also reduces the amplitude of the novelty P3a while increasing the amplitude of the salient P3b (27, 28). In contrast to healthy adults, acute nicotine treatment in adolescent females with MDD has been observed to reduce alpha power over the left hemisphere and increase power over the right hemisphere and central areas (29). However, there has been no examination of chronic nicotine treatment effects on EEG markers in MDD or LLD.

In this study, we evaluated EEG as a marker of treatment response in a small open-label pilot study of transdermal nicotine in adults with LLD. Patients with LLD completed an EEG recording at baseline and following 12 weeks of nicotine treatment. The present study included a subset of participants from a larger pilot trial of transdermal nicotine (30). We examined resting EEG activity pre- and post-nicotine intervention to assess changes to underlying frequency bands. Changes in attention were assessed using an auditory oddball task. We hypothesized that 12 weeks of nicotine treatment would result in a reduction of resting alpha power, and an increase in resting beta power over frontal regions. For the oddball task we examined the behavioral data and the event-related potentials and hypothesized that nicotine intervention would improve attention performance, resulting in greater accuracy and an increased parietal P300 peak for target compared to standard trials. We also examined event-related oscillations during the oddball task, to examine whether the nicotine intervention altered EEG power during attention task performance. Finally, an exploratory aim of the study was to determine whether changes in EEG were associated with changes in depressive symptoms.

## Materials and Methods

### Participants

Participants were recruited at Vanderbilt University Medical Center from clinical referrals and community advertisements from November 2016 through April 2017, with the study ending in August 2017. Core entry criteria focused on adults aged 60 years or older meeting DSM-IV-TR criteria for Major Depressive Disorder, recurrent or single episode, with a baseline depression severity measured by the Montgomery-Åsberg Depression Rating Scale [MADRS, (31)] of ≥15. Criteria related to cognitive function specified a Montreal Cognitive Assessment [MoCA, (32)] score of ≥24 but reporting subjective cognitive impairment, defined as endorsing ≥20% of items on the Cognitive Complaint Index [CCI, (33)]. Eligible participants could either be antidepressant-free or currently taking antidepressant monotherapy, however those taking antidepressants needed to be on a stable dose for at least 8 weeks. Additional exclusion criteria included: (1) Current tobacco or nicotine use in last year; (2) Other psychiatric disorders, except for anxiety symptoms occurring during a depressive episode; (3) History of alcohol or drug abuse over last 3 years; (4) Primary neurological disorders including dementia; (5) Regular use of drugs with centrally acting cholinergic or anticholinergic properties in the last 4 weeks; (6) Current psychotherapy. All participants provided written informed consent. The study was approved by the Vanderbilt University Medical Center Institutional Review Board. The study was registered with ClinicalTrials.gov (NCT02816138).

### Study Design

Participants were seen every 3 weeks plus a telephone call assessing tolerability at week 1. At each study clinic visit: (1) Depression severity was assessed by the study physician using the MADRS, (2) Subjective cognitive symptoms were assessed using the PROMIS (34), (3) Vital signs were assessed including sitting blood pressure, heart rate and weight, (4) Medication adherence was assessed using the Medication Adherence Questionnaire (35), and a patch count. EEG recordings took place at baseline (week 0) and following the completion of full dose nicotine treatment and before tapering (week 12).

### Study Drug Administration and Dosing

Transdermal nicotine was administered in a flexible dose escalation strategy with the ability to reduce to previous or intermediate doses for tolerability. The dose escalation strategy was: 3.5 mg (half of 7 mg patch) in week 1, 7 mg in weeks 2 and 3, 14 mg in weeks 4 through 6, and 21 mg in weeks 7 through 12. The target dose was 21 mg; however dose escalation or reductions were based on tolerability; participants were titrated to the highest dose that could be tolerated without side effects. If a participant had tolerability issues while using the 14 mg patch, the dose was initially reduced to an intermediate dose of 10.5 mg (half of 21 mg patch) and could be further reduced to 7 mg if needed. Participants were instructed to wear the study patch during the day and remove it at bedtime (~16 h daily); they were also instructed to move the patch location daily. Following trial completion, doses were tapered and discontinued over 3 weeks. Participants were seen at week 15 for a final visit.

### EEG Recording

EEG activity was recorded using a 128-channel Geodesic sensor net [EGI, Inc., Eugene, OR; (36–38)]. The EEG was sampled at 250 Hz with filters set at 0.1–100 Hz. During data collection, all electrodes were referenced to vertex (Cz). During the resting state recording participants had their eyes open and focused on a gray-fixation cross placed on a black screen. The resting state recording lasted for 3 min.

An auditory oddball task was performed to assess auditory attention (39, 40). A pair of two pure tones (single formant) at 1,000 and 1,500 Hz were the stimuli. Tones were equated in duration (300 ms) and rise/decay times. Tones were presented at 75 dB SPL (measured at the ear) through a speaker positioned 1 meter in front of the participant. A total of 200 trials were presented with the inter-stimulus interval varying randomly between 1,000 and 1,300 ms to prevent habituation to stimulus onset. The assignment of stimuli (high or low frequency) to standard and target conditions (70 and 30% of the trials, respectively) was counterbalanced across participants. Each participant was asked to press a different button using their preferred hand upon presentation of the standard and the target. On average, the auditory task lasted 6–7 min.

### EEG Processing

EEG data were processed using MATLAB (MATLAB, 2020) through a pipeline utilizing Fieldtrip (41), EEGLab (42), and CSD Toolbox (43) and in-house functions (A. Conley). Pre-processing was performed using Fieldtrip as follows. After being imported, EEG data was filtered using a high pass and notch filter to remove line noise and low-frequency drift (high pass: 0.1 Hz, forward phase; 60 Hz notch: zero phase). Excessively noisy channels were identified with visual inspection and excluded. For each oddball trial type (standard and target) epochs were extracted from −500 to 1,500 ms with respect to stimulus onset. For the resting state recording, the recorded data was broken up into 2,000 ms epochs. To remove blink and vertical eye-movement artifact, independent components analysis (ICA) was performed using the fastICA algorithm (44). This produces a set of components, 1 less than the number of available electrodes. Based on visual inspection by a trained observer, an average of 2.9 ± 0.5 components were removed that corresponded to ocular artifact (i.e., a deflection consistent with the time course of an eyeblink coupled with a frontal topographical distribution). The remaining components were projected back into sensor (electrode) space. The data were low pass filtered (30 Hz, zero-phase) to remove high frequency noise including muscular artifacts. Trials that contained residual artifact larger than ±150 μV were deleted. After artifact rejection, the surface Laplacian transformation of the EEG data was computed. For the surface Laplacian, a spherical spline function was applied across all scalp electrode locations, with the spline flexibility parameter, *m* = 4, for increased rigidness (45).

### ERP Analysis

Evoked potentials were extracted for standard and target trials in the oddball task over the midline parietal cortex. ERPs were baselined between −250 and −50 ms prior to stimulus onset. For the P300 waveform mean amplitude was extracted between 250 and 500 ms post-stimulus onset. The peak latency was extracted over this time window.

### EEG Power Analyses

**Figure 1:**
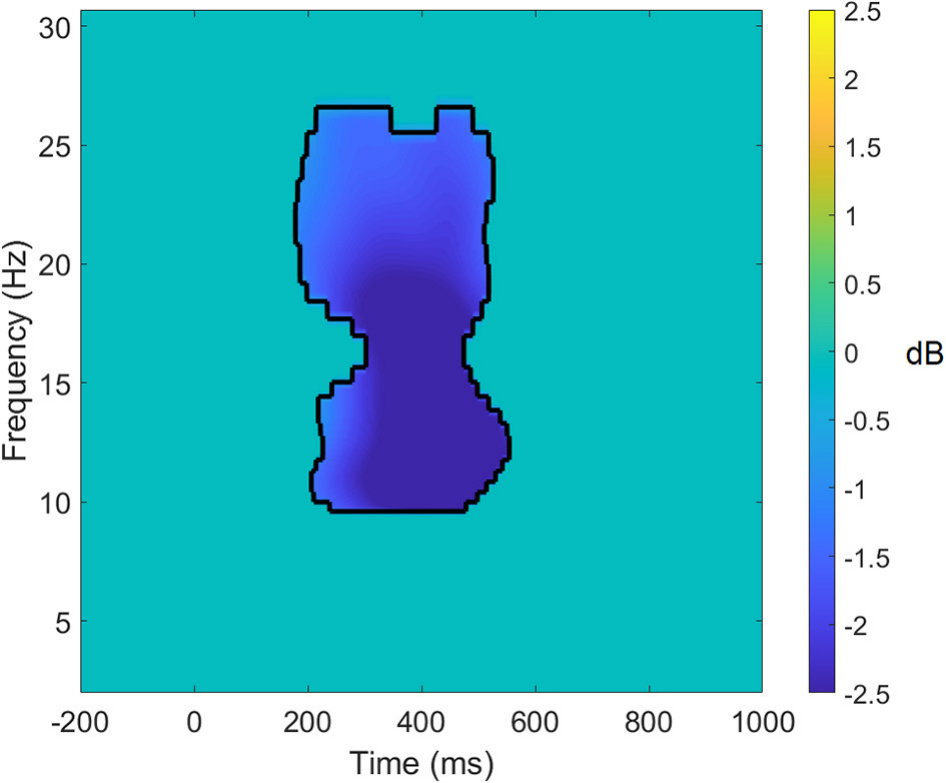
Significant target average data for the parietal cluster for the auditory oddball task. Data within the black lines represent frequency × time points that remain significant following false discovery rate correction of *p* < 0.01. All other data that does not survive the correction has been set to 0. Power differences are in dB.

EEG power was obtained by calculating the average of all decomposed single-trial time-frequency data using complex Morlet wavelets with 80 logarithmically scaled bins from 2 to 50 Hz (46). For the oddball task, power was calculated as a difference in respect to pre-stimulus baseline activity, which was defined as −250 to −50 ms prior to stimulus onset. No baseline correction was used to calculate the evoked power during the resting state recording. Significant changes in EEG power were identified using a process outlined by Cooper et al. (47). To identify significant and common changes in power from the pre-stimulus baseline, we combined data from both pre- and post-intervention sessions into a single task average for midline clusters at frontal, central and parietal locations [corresponding to Fz, Cz, and Pz according to the 10-10 system; (48)]. Following this, we performed one-sample *t*-tests at each frequency × time point for the three midline clusters for each of the tasks, with multiple comparison correction applied [false discovery rate, FDR *p* = 0.01; (49)]. Based on this analysis, we were able to identify common power processes associated with the tasks (see Figure 1). Next, we used these significant frequency × time clusters as masks and extracted average power for each of the four frequencies of interest (i.e., delta, theta, alpha, and beta) for each of the tasks (oddball and resting state) that were entered into the statistical analyses.

### Statistical Analyses

Changes in vital signs from Baseline to Week 12 were assessed using paired *t*-tests. Measurements of accuracy, response time, ERP and evoked oscillations derived from the oddball task were analyzed using a 2 × 2 repeated measures analysis of variance (ANOVA), with within-subjects' factors of time (baseline vs. week 12) and trial type (standard vs. target). Analysis of the different clusters (frontal, central, and parietal) of evoked EEG power were performed separately. Measures of resting EEG power were assessed by a 2 × 3 repeated measures ANOVA with within-subjects' factors of session and site (frontal, central, and parietal). Relation of evoked EEG activity to depressed symptoms (MADRS score) was completed by Pearson correlation. Statistical analyses were performed in jamovi stats version 1.2 (50).

## Results

### Participants

Fifteen participants were enrolled in the study, and 10 completed the EEG recordings. Markers of the safety and tolerability, as well as the clinical effectiveness of the nicotine treatment are reported in detail in Gandelman et al. (30). One participant did not complete their follow-up visit, and one participant had to be excluded due to EEG artifacts. Average trial numbers for the participants that were included in the analysis for the oddball task were for Baseline: 62.9 ± 26.7 standards and 24.8 ± 12.6 targets, and for Week 12: 68.4 ± 25.9 standards and 31.5 ± 5.3 targets. The demographic information of the final sample of 8 participants is described in Table 1. All 8 participants in the final sample were Caucasian.

**Table 1:** Demographic information.

**Measure**	**Total group**	**Augmentation**	**Monotherapy**
	**(*n* = 8)**	**(*n* = 5)**	**(*n* = 3)**
Age (yrs)	64.8 (5)	66.4 (5.9)	62 (1)
Sex, women, *N* (%)	5 (62.5%)	4 (80%)	1 (33%)
Education (yrs)	17.13 (1.4)	16.8 (1.5)	17.7 (1.2)
Past smoker, *N* (%)	6 (75%)	4 (80%)	2 (67%)
Age at first depressive episode (yrs)	29.3 (20)	35.4 (23.6)	19 (5.3)
Duration of current episode (days)	891 (698)	1,184 (710)	401 (350)
MoCA at Baseline	27.8 (0.9)	27.8 (1.1)	27.7 (0.6)
MADRS at Baseline	28.1 (7.1)	28.8 (5.5)	27 (3.6)
MADRS at Week 12	11.1 (9.6)	10.6 (10.7)	12 (9.5)
Maximum Nicotine Dosage (mg)	16.63 (6.1)	21 (0)	9.33 (2)
**Psychotropic Medication**, ***N***
Sertraline	2	2	0
Venlafaxine	2	2	0
Duloxetine	1	1	0
Trazadone	1	1	0
Lorazepam	1	1	0

*Data presented as mean (SD) unless specified. Past smoker is defined as having smoked a cigarette daily for at least 6 months over the participant's lifetime. MADRS, Montgomery-Asberg Depression Rating Scale; MoCA, Montreal Cognitive Assessment*.

### Tolerability of Nicotine Intervention

No changes in blood pressure or pulse were reported across the 12 weeks of treatment. There was a significant decrease in body weight (mean decrease: 3.57 kg, *t* = 3.2, *p* = 0.015, *d* = 1.14), and BMI (mean decrease: 1.04, *t* = 3.25, *p* = 0.014, *d* = 1.15) for participants from Baseline to Week 12. The mean final patch dose was 16.63 mg (SD = 6.1 mg, range 7–21 mg) with 5 achieving the maximum 21 mg dose. Participants exhibited >90% medication adherence on average with study patches.

### Auditory Oddball Results

#### Behavioral Results

The results of participants on the auditory oddball task are reported in Table 2. Participants' accuracy performance on the oddball task remained stable across the 12-week intervention (Baseline: 81 ± 16.1%; Week 12: 84 ± 11.1%), and there was no effect of either time or trial type (all *p* > 0.3). However, participants responded significantly faster after nicotine treatment compared to baseline [488 ± 28.6 vs. 434 ± 20.4 ms, *F*_(1, 7)_ = 12.5, *p* < 0.005, $\eta_{\text{p}}^{2}$ = 0.64], with the greatest improvement from baseline to post-intervention being in response to targets compared to non-target standards [Standard: 474 ± 82.4 vs. 434 ± 64.6 ms; Target: 502 ± 79.4 vs. 434 ± 50.9 ms; Trial × Session: *F*_(1, 7)_ = 6.4, *p* = 0.04, $\eta_{\text{p}}^{2}$ = 0.48].

**Table 2:** Auditory oddball results.

	**Baseline**			**Week 12**		
	**Overall**	**Standard**	**Target**	**Overall**	**Standard**	**Target**
Accuracy (%)	81.3 (16.1)	83.8 (15.7)	78.9 (17.9)	84.4 (11.1)	84 (15.1)	84.8 (8.6)
Response time (ms)	488 (80.4)	474 (82.4)	502 (79.4)	434 (55.7)	434 (64.6)	434 (50.9)
P300 amplitude (μV)	1.8 (2.2)	1.2 (1.8)	2.4 (2.5)	0.3 (3.1)	−0.5 (2.7)	0.99 (3.6)
P300 peak latency (ms)	412 (54)	402 (50)	421 (58)	385 (61)	373 (61)	396 (62)
**Delta Power (dB)**
Frontal	1.5 (1.2)	0.76 (0.77)	2.17 (1.9)	1.02 (0.9)	0.28 (1.1)	1.77 (1.3)
Central	1.4 (0.7)	0.46 (0.56)	2.3 (1.2)	1.2 (1)	0.78 (1.2)	1.7 (1.2)
**Alpha Power (dB)**
Central	−1.05 (0.7)	−0.4 (0.17)	−1.7 (1.2)	−1.1 (0.65)	−0.37 (0.2)	−1.9 (1.1)
Parietal	−1.1 (0.84)	−0.4 (0.26)	−1.7 (1.5)	−1.1 (0.64)	−0.35 (0.24)	−1.8 (1.1)
**Beta Power (dB)**
Frontal	−0.46 (0.3)	−0.35 (0.2)	−0.57 (0.45)	−0.54 (0.4)	−0.5 (0.3)	−0.58 (0.5)
Central	−1.7 (0.8)	−1.1 (0.6)	−2.4 (1.2)	−1.95 (1.1)	−1.4 (0.7)	−2.6 (1.5)
Parietal	−0.85 (0.5)	−0.48 (0.3)	−1.23 (0.74)	−0.6 (0.43)	−0.34 (0.24)	−0.86 (0.7)

*Data presented as mean (SD). Frontal, central and parietal refer to midline clusters. P300 amplitude and latency are from the parietal cluster. Power statistics refer to the magnitude change post-stimulus compared to pre-stimulus baseline. Overall refers to task average performance of standard and target trials*.

#### Event-Related Potentials

As shown in Table 2, the P300 amplitudes decreased for both oddball and standard trials after the 12-week intervention, however the decline in amplitude was larger for standard trials (1.6 vs. 1.4 μV). The latency of the P300 evoked by participants decreased for both trial types by between 20 and 30 ms (29 vs. 25 ms) following treatment. Analysis of the ERP markers of mean amplitude and the peak latency of the parietal P300 showed no effect of time, trial type or an interaction between the two factors (all *p* > 0.05).

#### Event-Related Power

The results of the analysis of the task-averaged power evoked at the frontal cluster revealed a mask of significant time × frequency values in the delta band between 400 and 800 ms and the beta bands between 200 and 500 ms. For the central cluster, the mask showed significant time × frequency values in the delta band between 400 and 600 ms, in the alpha band between 200 and 400 ms, and in the beta band between 200 and 600 ms. In the parietal cluster, the task-averaged data showed significant differences from zero in the alpha and the beta bands between 200 and 500 ms post-stimulus.

The results of the repeated measures ANOVA for the frontal cluster identified a significant main effect of the trial type in the delta band, with greater evoked delta power for targets compared to standards [0.52 ± 0.9 vs. 1.97 ± 1.6 dB; *F*_(1, 7)_ = 8.5, *p* = 0.02, $\eta_{\text{p}}^{2}$ = 0.55]. However, there was no difference in the evoked delta power between baseline and Week 12 (*F* <1). There was also no difference in the evoked beta power at the frontal cluster (all *p* > 0.2).

As with the frontal cluster, the ANOVA of the central cluster revealed large differences between trial types in all frequency bands (all *p* < 0.005). This trial effect evoked greater synchronization of delta power, and greater desynchronization of alpha and beta power to targets compared to standards. However, there was no impact of the nicotine treatment on power in the delta, alpha or beta bands.

**Figure 2:**
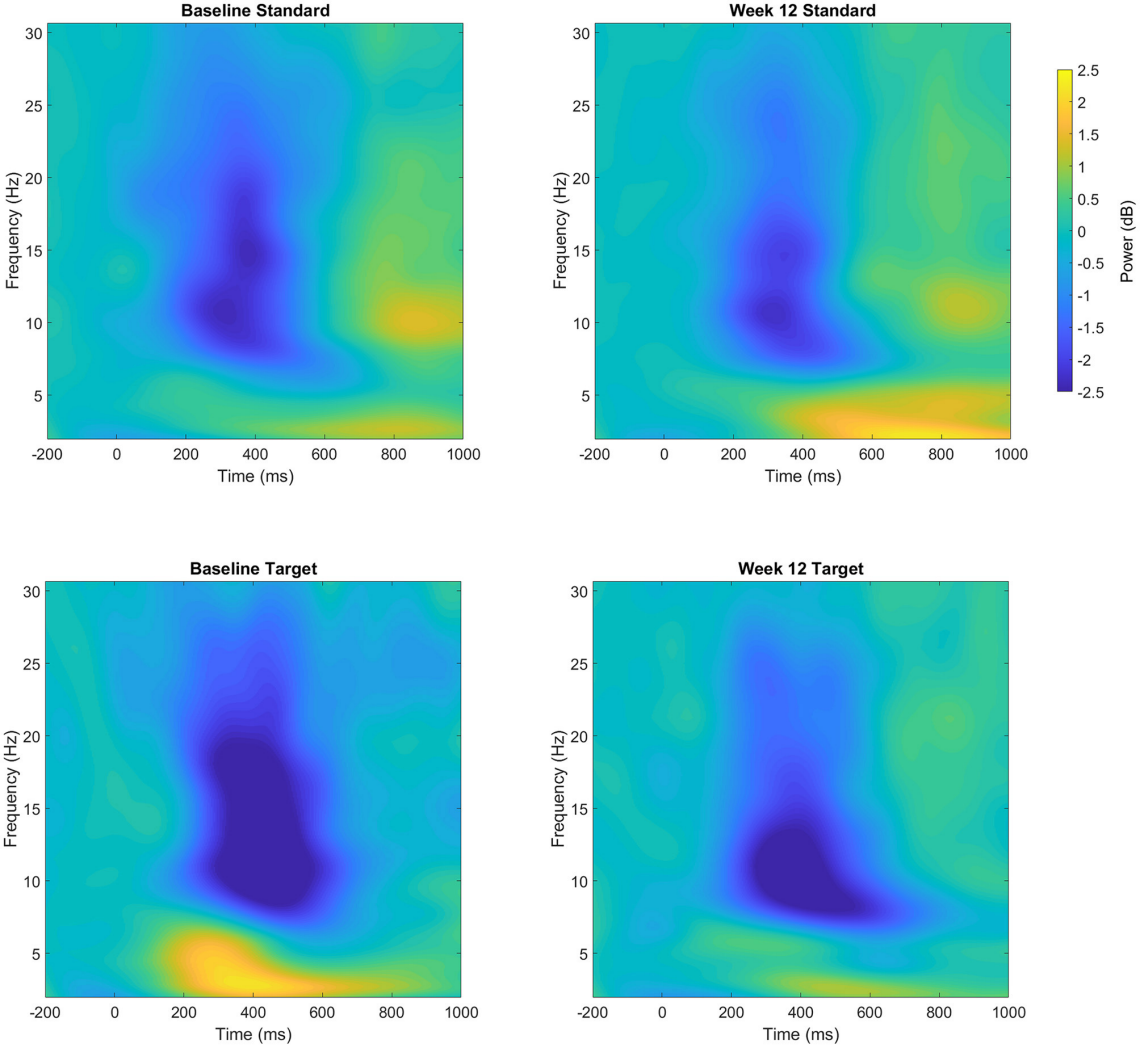
Event-related power (dB) at both Baseline and Week 12 following standard (top row) and target (bottom row) trials over the parietal cluster. Changes in power reflect increases or decreased magnitude compared to the pre-stimulus baseline.

The analysis of the parietal cluster (see Figure 2) showed a significant main effect of the trial type in the alpha band, with greater desynchronization of alpha power for targets compared to standards [−0.39 ± 0.25 vs. −1.77 ± 1.3 dB; *F*_(1, 7)_ = 14.2, *p* < 0.005, $\eta_{\text{p}}^{2}$ = 0.67]. There was however no difference in the evoked alpha power between baseline and Week 12 (*F* <1). The analysis of the beta band showed that following 12 weeks of nicotine treatment, there was a reduction in the desynchronization of beta over the parietal cluster [Baseline: −0.85 ± 0.5 vs. Week 12: −0.6 ± 0.45 dB; *F*_(1, 7)_ = 11.1, *p* = 0.013, $\eta_{\text{p}}^{2}$ = 0.61]. This shift in beta power across the 12-week treatment period was greater for target trials compared to standard trials [mean difference 0.37 vs. 0.14 dB; Trial × Session: *F*_(1, 7)_ = 5.4, *p* = 0.05, $\eta_{\text{p}}^{2}$ = 0.44].

### Resting State EEG Results

The mean resting power at baseline and at week 12 is displayed in Table 3. The results of the session average resting data revealed significant time × frequency differences from 0 for all frequency bands at each of the three midline clusters. However, the analysis of the midline clusters did not show any change in resting power following nicotine intervention for any of the frequency bands (all *p* > 0.1). There was a significant difference in resting beta power across the sites, which showed a reduction in beta power from frontal to central clusters, with the lowest power at the parietal cluster [*F*_(2, 14)_ = 3.96, *p* = 0.044, $\eta_{\text{p}}^{2}$ = 0.36]. However, this difference in resting power across the scalp did not change with nicotine treatment (*p* = 0.3).

**Table 3:** Resting EEG power at baseline and week 12 at midline clusters.

**Power (dB)**	**Baseline**			**Week 12**		
	**Frontal**	**Central**	**Parietal**	**Frontal**	**Central**	**Parietal**
Delta	57.8 (2.4)	56 (1.8)	55.1 (2.5)	57.5 (1.6)	56 (2.6)	56.9 (3.5)
Theta	51.7 (2.2)	50.9 (2.2)	49.9 (3.1)	51 (1.7)	50.3 (2.7)	50.6 (3.7)
Alpha	47.9 (2.5)	47.9 (3.6)	50 (4.3)	47.1 (2.0)	47.9 (3.6)	49.5 (4.5)
Beta	44.4 (3.7)	43 (3.0)	42.5 (2.7)	43.6 (2.3)	43.6 (3.8)	42.6 (2.95)

*Data presented as mean (SD). Power represented in the table is absolute power at each cluster*.

### Relationship to Depressive Symptomatology

**Figure 3:**
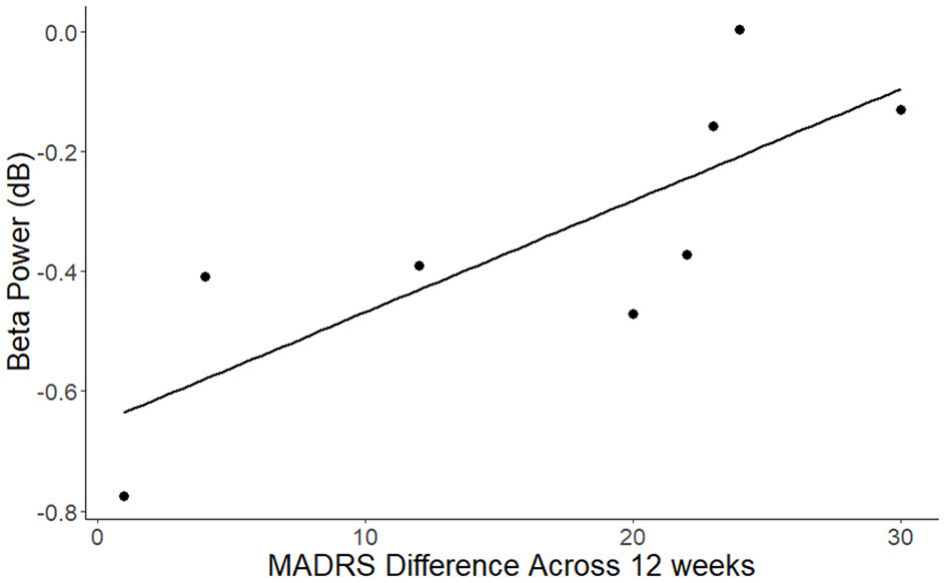
Association between change in MADRS scores across the nicotine treatment period and beta power for standard trials over the parietal cluster (*r* = 0.8, *p* < 0.02). Beta values reflect decreases compared to the pre-stimulus baseline.

Analysis of the relationship of improvement in depression score and EEG/ERP signals following nicotine treatment showed that the reduction in MADRS scores after nicotine treatment was significantly correlated with the post-intervention parietal beta power for standard tones (Figure 3; *r* = 0.8, *p* < 0.02). This relationship reflected that greater improvement in MADRS scores following nicotine treatment were associated with a greater reduction of parietal desynchronization to standard trials. The reaction time performance of the oddball task or the resting beta power did not correlate with the change in depressive symptoms across the nicotine treatment period (all *p* > 0.1).

## Discussion

This pilot study showed that 12-week treatment of open-label transdermal nicotine produced improved attention performance in patients with LLD. This functional engagement of the nicotinic system was evident in reduced reaction time and the reduction of beta desynchronization during the oddball task following nicotine treatment. The change in parietal beta power was correlated with mood improvements on the MADRS.

Twelve weeks of transdermal nicotine resulted in improved performance on the oddball task, although not as we had hypothesized. While we had predicted improved accuracy and increased amplitude of the target P300, however compared to baseline, neither of these measures changed across the intervention period. Instead, participants exhibited faster reaction time, with the enhancement being greater for targets compared to standards. Consistent with the reaction time changes, desynchronization of beta power over the parietal cortex was reduced for both target and standard trials following nicotine intervention, with greater desynchronization for target trials. Moreover, the changes in beta oscillations for the standard trials was also correlated with the change in MADRS scores, with greater beta power corresponding to a greater reduction in depressive symptoms. While this was an open-label trial, and therefore we cannot rule out the change in oddball performance to be related to time or practice, the finding that the changes in evoked oscillations were associated with a positive change in MADRS scores across the intervention period suggests that we observed functional engagement of the cholinergic system, resulting in improved attention performance which correlated with improved mood. Beta oscillations are commonly associated with visual attention and motor performance (51). In addition, cholinergic stimulation, in this case through transdermal nicotine, is known to modulate arousal and attention (20, 52). Therefore, this finding suggests that observed a reduction of parietal beta band desynchronization following nicotine is related to an improvement of attention performance, through the reduction of task-irrelevant activity, resulting in improved performance on the oddball task. In contrast to prior studies and to our results from the oddball task, we did not see a significant change in resting EEG power across the nicotine treatment period.

We did not see changes in the alpha band. Attention in healthy adults has been also related to oscillations in the alpha band, and specifically a desynchronization over the parietal cortex (53). Alpha synchronization is associated with inhibition of activity (54, 55), and also mental fatigue and drowsiness (56, 57). Desynchronization of the alpha band is associated with temporal attention and sensory gating (58, 59). Specifically in auditory tasks, alpha oscillations are thought to drive attention allocation in the cortex (60, 61). Therefore, the fact that task-relevant alpha desynchronization was not substantially altered across treatment may reflect efficient processing or may be a result of the age-related slowing of the EEG spectra, whereby across healthy aging, the power of faster frequency bands decreases, while the slower power bands increase in power (62). Slowing of the spectra is increased in clinical populations including those with MCI (63). Thus, a change in beta power following treatment may be more noticeable in older adults, due to reduced power compared to the slower frequency bands.

Depression is associated with deficits in attention processes (64, 65). These deficits in attention have downstream effects on several higher order cognitive processes including executive functions (66). Attention itself has been characterized as having four hierarchical subdomains, including intrinsic alertness, sustained attention, focused or selective attention and behavioral inhibition (67, 68). Compared to healthy controls, adults with MDD show deficits on selective attention (69, 70), and sustained attention (71, 72). Within the context of the present study, the results indicate that nicotine may be enhancing sustained attention. While there was not a substantial change in accuracy across the 12 weeks, responses were faster following treatment. There may also be an impact of the improved mood, as evidenced by a decline in MADRS scores, on attention. Positive mood has previously been reported as an enhancer of other cognitive processes (73–75). For auditory attention, recent studies have shown that in healthy controls improving mood through music or mindfulness enhances performance to auditory stimuli (76, 77). As such, the improvement of attention by cholinergic stimulation, and the enhancement of mood may be additive factors for cognitive processing.

The finding of significant oscillatory changes in the parietal cortex may indicate that the cholinergic stimulation is improving frontoparietal connections in adults with LLD. Healthy aging is associated with a shift in functional activity from parietal to frontal regions (78, 79), but this shift is exacerbated in the presence of Alzheimer's pathology (80, 81) and also depressive symptoms (82, 83). As the parietal cortex is important for both auditory and visual attention (53, 84–86), the posterior-anterior shift in processing results in a reorganization of cortical activation for attention tasks (78). This is observed in healthy older adults, with P300 amplitudes shifting from parietal to frontal areas (87, 88). Adults with MDD show a reduction in frontoparietal connectivity during both visual and auditory attention tasks (89–91). In the context of the present study, cholinergic stimulation through nicotine treatment may be stimulating activity in the less functionally active areas of the parietal cortex during attention tasks. This activation during attention tasks may promote increased connectivity within the frontoparietal control network, and lead to downstream improvements in cognitive control in adults with LLD.

In contrast to previous studies, we did not find changes in resting oscillatory activity following nicotine treatment. This may be due to the differences in age or the focus of the analysis on midline clusters rather than looking at hemispheric differences. As discussed above, Jaworska et al. found an increase in global alpha power over the right hemisphere in young women with MDD following acute transdermal nicotine (29). Lateralization of brain activity have been reported consistently in younger adults with MDD (92) however, this finding has not been reliably found in adults with LLD (93). Future investigation would be needed to confirm whether functional lateralization is seen following nicotinic treatment in adults with LLD.

A limitation of this pilot study is that the nicotine was administered to patients in an open-label design, and as such there was not a placebo group to compare the effects across the 12-week period. The sample of participants was uniformly Caucasian, which does limit the generalizability of the results. The sample size of the trial was also small, which limits the ability to test for dose effects, and reduced overall power to detect changes in ERP or resting state activity. Despite this, the pilot study identifies EEG markers of activity that are both indicative of treatment effect and relate to a change in depressive symptoms.

## Conclusion

In this pilot study we show that 12 weeks of transdermal nicotine treatment can enhance event-related oscillations associate with auditory attention in adults with LLD. Moreover, this change in cortical activity was related to the magnitude of improvement in mood symptoms over the course of the nicotine treatment, which highlights the potential use of EEG marker for predicting treatment response in adults with LLD. Future studies should aim to confirm this finding in larger, placebo-controlled trials which would be able to conclusively show the efficacy of an investigational treatment.

## Data Availability Statement

The raw data supporting the conclusions of this article will be made available by the authors, without undue reservation.

## Ethics Statement

The studies involving human participants were reviewed and approved by Vanderbilt University Medical Center Institutional Review Board. The patients/participants provided their written informed consent to participate in this study.

## Author Contributions

AC contributed to the design of the experiment, processing of EEG data, and the design and preparation of the manuscript. AK contributed to the design of the procedures and the preparation of the manuscript. WT contributed to the design of the experiment and analyses, data collection and processing, and preparation of the manuscript. KA, BB, and JV contributed to the design of the preparation of the manuscript. PN contributed to the design of the experiment and analyses and the preparation of the manuscript. All authors contributed to the article and approved the submitted version.

## Funding

This research was supported by NIH Grant K24 MH110598 and CTSA Award UL1TR000445 from the National Center for Advancing Translational Sciences.

## Conflict of Interest

The authors declare that the research was conducted in the absence of any commercial or financial relationships that could be construed as a potential conflict of interest.

## Publisher's Note

All claims expressed in this article are solely those of the authors and do not necessarily represent those of their affiliated organizations, or those of the publisher, the editors and the reviewers. Any product that may be evaluated in this article, or claim that may be made by its manufacturer, is not guaranteed or endorsed by the publisher.
